# Sex and Gender Disparities in Kidney Transplantation

**DOI:** 10.1053/j.akdh.2025.01.003

**Published:** 2025-05

**Authors:** Mythri Shankar, Silvi Shah

**Affiliations:** From the Department of Nephrology, Institute of Nephro-urology, Bengaluru, India (M.S.); and Division of Nephrology and Hypertension, Department of Internal Medicine, University of Cincinnati, Cincinnati, OH (S.S.).

**Keywords:** Sex, Gender, Kidney transplant, Deceased donor, Living donor

## Abstract

Sex refers to biological traits, while gender involves socially constructed roles and behaviors. Globally, women face disparities in access to kidney transplants, and outcomes, driven by sociocultural and systemic factors. Females have a higher prevalence of kidney disease, start dialysis at lower glomerular filtration rates, and receive lower dialysis doses than males. Fewer females are refered for kidney transplants than males, and females have lower rates of preemptive transplantation than males. There are significant disparities in waitlisting, with fewer females being placed on kidney transplant waiting list and females having longer median wait time, as compared to males. Studies indicate variable outcomes in kidney allograft survival in males and females due to differences in immune response, hormonal effects, and nephron mismatch. Socioeconomic, cultural, and psychosocial factors exacerbate these gaps, alongside healthcare provider biases. Females constitute over 75% of donors, often reflecting caregiving roles. Addressing these disparities requires equitable organ allocation systems, strengthened donor exchange programs, financial support, and culturally sensitive education. Research and policy reforms remain critical to bridging the sex and gender gap in kidney transplantation.

## DEFINITION OF SEX AND GENDER

Sex typically refers to a set of biological characteristics linked to physical and physiological traits, such as chromosomal genotype, hormone levels, and internal and external anatomy. In contrast, gender pertains to the socially constructed roles, behaviors, and identities of women, men, and gender-diverse individuals within historical and cultural contexts. Biological differences between males and females due to hormones, genetics, and anatomical factors may influence the disease phenotype; similarly, behavior and cultural factors may influence disease identification, treatment-seeking behavior, compliance, and dealing with the disease per se. Hence, both sex and gender influence the epidemiology, natural history, and disease outcomes.^1^

Global Perspectives of Sex and Gender Disparities in Kidney Transplantation

The global prevalence of CKD and kidney failure is rising, resulting in parallel increase in the need for kidney transplantation. Sex and gender disparities in access to kidney transplantation, rates of kidney transplantation, patient and kidney allograft outcomes are prevalent worldwide. A multicountry research study including Germany, France, Brazil, and the United States (CKDopps) reported that women exhibited lower rates of initiation on dialysis and pre-emptive transplantation than men. Notably, France diverged from this trend, showing no significant differences between sexes in pre-emptive transplantation rates or the number of waitlisted patients. Generally, men were more likely to have vascular access established before the initiation of dialysis and were more frequently started on hemodialysis. In contrast, peritoneal dialysis was marginally more prevalent among women in France, Brazil, and Germany, but not in the United States. These varying outcomes across different countries suggest that nonbiological factors, such as sociocultural and systemic influences, may substantially shape health care decisions.^2,3^

In Mexico, sex disparity was also observed among kidney transplant recipients (62.4% male vs 37.6% female), with majority of kidney transplant recipients being males. Additionally, the rate of female kidney transplant recipients steadily declined from 40.7% to 35.8% over a period starting from 2007 until 2019. The study also reported a similar trend in living kidney donors, with a higher proportion of living kidney donors being females compared to males (53.1% vs 46.9%).^4^

Recent research conducted by the Asian Society of Transplantation, in collaboration with Women in Transplantation, examined sex disparities in kidney transplantation across the Asia-Pacific region. They revealed that the proportion of female living kidney donors was significantly more than that of male donors, except for Hong Kong, Pakistan, and the Philippines. Remarkably, the proportion of female spousal donors significantly exceeded that of male spousal donors, with rates varying from 64% to 90% across the Asia-Pacific region.^5^ Similar trends were observed in China, where most living kidney donors were females compared to males (69.2% vs 30.8%), while most recipients were males (79.5% vs 20.6%). These disparities could be due to socio-cultural and financial factors, indicating the outcomes could be a result of health care decisions and reduced access to care.^6^

A recent survey of Indian nephrologists concerning gender-based access to kidney transplants revealed significant disparities. Among the 267 respondents, 80% indicated that women constituted less than 25% of kidney transplant recipients and over 75% of donors in their practice. Women received less kidney replacement therapy or kidney transplants compared to men. This survey identified several factors contributing to the disparity in access to transplantation: family members were unwilling to donate to female recipients (64%), women were reluctant to accept a kidney from a family member (44%), misconceptions about transplantation (16.5%), and financial barriers (36%). Furthermore, the survey revealed a significant difference in donor relationships: when women were the recipients, 85% of the donors were parents, whereas when men were the recipients, approximately 70% of the donors were spouses.^7^

### Sex and Gender Disparities in Kidney Transplant Referral and Evaluation

Men are more frequently initiated on kidney replacement therapy despite the higher prevalence of CKD among women.^8^ Several hypotheses are proposed to account for the slower progression of CKD in women. These include potential inaccuracies in estimated glomerular filtration rate calibration, the nephroprotective effects of female hormones, and the detrimental impact of testosterone, as evidenced in experimental studies. Additionally, a higher prevalence of unhealthy lifestyle behaviors among men compared to women has also been suggested as a contributing factor. The most common etiologies for end-stage kidney disease are diabetes mellitus and hypertension for males, and additionally, autoimmune diseases such as lupus nephritis and pregnancy-related kidney diseases are common in females.^9^ This is a significant point of discussion, as the initiation of kidney replacement therapy commonly triggers referral for transplant evaluation.^10^ Additionally, women tend to start dialysis at slightly lower estimated glomerular filtration rate levels and receive lower dialysis doses compared to men.^11^

The latest United States Renal Data System (USRDS) data indicates that there is no significant difference between men and women receiving CKD care.^12^ Despite identical CKD care, a significant disparity exists in access to treatment in patients with kidney failure, including kidney replacement therapy and kidney transplants. Recent years have seen limited research into gender-specific differences in referral patterns; however, one study investigated kidney transplantation referrals and the initiation of the evaluation process among dialysis patients.^13^ The results showed that the median referral rate within 1 year was 33.7% (ranging from 0% to 100%). Nevertheless, less than 50% of these referred patients began their evaluation process in less than 6 months, representing only 16.1% of all new dialysis patients. The study also identified factors such as insurance, older age, females, and neighborhood poverty as the probable reasons for lower referral and transplantation evaluation rates. These findings highlight persistent barriers postreferral, which remain a critical area seeking attention. The policy targeting these disparities in the United States is the “Advancing American Kidney Health” Executive Order (2019).^14^ To take care of this issue, the Centers for Medicare and Medicaid Services will align payments to facilities providing dialysis and treating nephrologists based on the rates of home dialysis and kidney transplantation, promising to enhance access to transplantation while interventions are required to mitigate other barriers.

### Sex and Gender Disparities in Kidney Transplant Waitlisting

A comparative analysis of the United States of America and Austria indicates sex disparities in both countries. Both have a higher number of males compared to females listed for kidney transplants. However, there are indications of a shift toward a better equitable process in other aspects of kidney transplants, demonstrated by a gradual rise in the number of male living donors and female transplant recipients.^15^ Though there has been progress toward gender equity in kidney transplantation reported in recent studies, the majority of data from other research indicate that sex disparities do persist, particularly in access to the waitlist. A study from the United States demonstrated that females had 11% less access to kidney transplant waiting list compared to males after adjusting for the confounding variables. Especially affected were females older than 65 years of age.^16^ A French study showed that women spent longer time on dialysis compared to men who got transplanted early, despite universal access to health care. Particularly affected were elderly females, financially dependent females, and diabetic females.^17^ Another research study from Germany supported the findings and showed that females had 18% lower access to the kidney transplant waitlist compared to males.^18^ Subsequently a study from United Kingdom demonstrated that the recipient sex was no longer a significant factor for disparity to access the waitlist, other factors that influenced were elderly age, Africans, Hispanics, Asians, and lower socioeconomic status.^19,20^

### Sex and Gender Disparities in Kidney Allograft Outcomes and Patient Survival (Table 1)

#### Graft and Survival Variability.

Several studies indicate differing outcomes between male and female recipients:

- Females exhibit a more robust immune response, potentially leading to higher rates of acute rejection and lower graft survival in some cases.^21-23^
- Conversely, studies suggest that the sex of the deceased donor (male vs female) does not significantly impact graft survival, although there is a noted variance in primary nonfunction and delayed graft function rates based on donor sex.^31^

#### Influence of Donor-Recipient Sex Matching.

- The impact of donor-recipient sex mismatch has been extensively debated:
  - Analysis from the Collaborative Transplant Study noted that female recipients of male donors showed the best allograft and patient survival, while male recipients of female donors exhibited the worst outcomes.^26^
  - This could be due to the lower nephron mass in female donors compared to the elevated functional demands of male recipients, suggesting a physiological mismatch that affects graft survival.^27^
  - Additionally, female recipients of male donor kidneys showed a higher risk of graft failure and increased mortality rate within the first year post-transplant, though this risk disparity diminishes over a longer period.^23^
  - Short-term graft survival is reportedly poorer in female recipients compared to males, yet long-term survival rates are better for females, particularly among those older than 45 years.^28,29,32^

A meta-analysis showed that compared to male recipients, female recipients exhibited poor short-term graft survival and good long-term graft survival.^23^ Similarly, an analysis of USRDS data revealed that females had a 10% greater risk of acute rejection in the initial 6 months of post-transplant period but a 10% lower risk of long-term graft loss due to chronic allograft injury, particularly among patients who were older than forty-five years of age.^28^ A study analyzing data from the USRDS on deceased donor kidney transplants found that female recipients of male donor kidneys exhibited a 12% higher risk of graft failure and an elevated mortality rate 1 year post-transplant. However, this increased graft failure or mortality risk was not observed in female recipients 10 years post-transplant.^29^ This study showed that the graft failure rates were higher in female recipients who received kidneys from male donors, probably due to the H–Y minor histocompatibility antigen.^29^ Analysis of the Scientific Registry of Transplant Recipients data involving more than 150,000 kidney transplant recipients revealed significant sex-based differences in graft failure risks. Among recipients of male donor kidneys, females of all ages exhibited a significantly higher risk of graft failure compared to males. In contrast, for recipients of female donor kidneys, the risk of graft failure varied by age: females aged between 15 and 24 years faced a higher risk, whereas females aged 45 years and older faced a lower risk than their male counterparts. Both innate and adaptive immunity are influenced by age and sex. Post-puberty, women have a robust immune system under the influence of estrogen; on the other hand, androgen has immunosuppressive effects. This predisposes men to infections and menstruating women to increased rejection rates and graft failure, which reduces drastically postmen-opause.^33^

These sex differences in graft outcomes are influenced by multiple factors, such as age-related immune responses, the effect of sex hormones on the immune system, genetic factors, compliance patterns, variations in body size and nephron mass, and sex-related minor histocompatibility antigens. Females have higher immune reactivity due to greater X chromosome gene expression and HY antigen histocompatibility. Pharmacokinetics of immunosuppressive medications also play an important role; for example, calcineurin inhibitors have lower trough levels in females, making them susceptible to rejection. Unfortunately, many drug trials exclude females of reproductive age group; hence these discrepancies are not identified earlier.^34^

A study involving over 25,000 deceased donor kidney transplant recipients demonstrated similar age-associated outcomes when studied for sex differences. They found no overall difference in graft survival between the sexes but observed age-specific variations: female recipients aged 25 to 44 years had significantly shorter graft survival rates when compared to males, whereas this trend reversed in recipients aged 45 years and older.^32^ A study was conducted to assess the relation between allograft loss and recipient socioeconomic status. The results showed that women from low- to middle-income neighborhoods had a significantly higher risk of graft loss compared to men after living donor kidney transplants. Conversely, women from middle- to high-income neighborhoods demonstrated better and more equitable patient survival rates than the male counterparts. This study underscores the huge impact of socioeconomic status on women’s health, particularly regarding kidney transplant outcomes.^30^

### Sex and Gender Disparities in Living Donor Kidney Transplantation

Living donor kidney transplantation is the optimal therapy for patients with kidney failure who are eligible for transplant. However, recent study indicates a 30% lower rate of living donor kidney transplantation for women compared to men. Women lag behind men specifically at the human leukocyte antigen testing and breast malignancy and other gynecological malignancies evaluation stage of the living donor kidney transplantation process. The incompatibility rate due to sensitization from prior blood transfusions or transplants was similar for both genders. However, women with a history of pregnancy exhibited significantly higher rates of living donor incompatibility, posing a considerable challenge given that a significant proportion of potential donors for these women are their spouses or offspring. This disparity is further exacerbated by the fact that women, despite constituting 63% of the living donor pool, are less likely to receive a kidney from a potential living donor.^35,36^

Kidney paired donation is a strategy that enables incompatible donor-recipient pairs to exchange kidneys with other incompatible pairs, resulting in two compatible living donor kidney transplants. This approach significantly enhances kidney transplant opportunities for many patients with kidney failure, particularly among racial minorities and sensitized women. Increased participation as well as implementation of kidney paired donation programs are the need of the hour for these group of patients.^35,37^

### Sex and Gender Disparities in Hepatitis C Virus Viremic Donors

Over the past decade, a significant advancement in mitigating the organ shortage has been the transplantation of kidneys from hepatitis C virus (HCV) viremic donors into HCV-negative recipients (HCV D+/R−), followed by direct-acting antiviral therapy. Early data indicate promising outcomes, such as shorter waitlist times and access to younger donors with excellent allograft function.^38^ However, a study examining the effects of this new donor pool on racial minorities and women found that these groups remain disadvantaged. Specifically, women were 20% less likely to receive a kidney from HCV-positive donors, suggesting potential conscious or unconscious biases in patient selection by transplant centers.^39^ Educating patients about HCV-positive donors requires considerable time and effort, which may have been disproportionately directed toward patients perceived as more likely to benefit from this donor source. Alternatively, more culturally competent education tailored to the specific needs of women may be necessary to address this disparity.

### Sex and Gender Disparities Pediatric Transplantation

A European study encompassing 35 countries revealed that while the overall transplantation rates have been comparable between boys and girls, the pre-emptive transplant rates were found to be 23% lower in girls compared to boys, resulting in prolonged durations on dialysis. This disparity could not be attributed solely to medical factors; instead, attitudes or biases of the health care providers and parents might be the likely contributors.^40^ Additionally, it was found that girls were less frequently waitlisted for deceased donor transplants than boys.^40^

### Role of Biological Factors in Sex and Gender Disparities in Kidney Transplantation

Biological factors are one of the major contributors to sex disparity in kidney transplantation. Males typically experience a rapid progression of kidney disease, leading to earlier referrals for kidney transplantation. Females, on the other hand, may be prone to higher levels of sensitization and formation of H–Y antibodies, most probably due to pregnancy, which reduces their likelihood of matching with an organ donor, particularly from male partners or spouses.^41,42^

Along with the standard pretransplant or predonation risk assessments, females must undergo screening for gynecologic malignancies and breast cancer. This additional screening requirement can delay female recipients or donors’ evaluation and acceptance process. Comorbid conditions such as pre-exiting kidney disease or cardiovascular disease, which are more prevalent in males, also reduce their eligibility as organ donors. In premenopausal female recipients, graft outcomes might be negatively impacted by the estrogen-signaling effects on the immune system. Estrogen promotes proinflammatory cytokine production, B-cell survival and maturation, and toll-like receptor expression, causing a stronger T-cell activation and potentially increasing the rates of acute allograft rejection. Additionally, female transplant recipients who care for young children are at an increased risk for post-transplant infections. Women are also at an increased risk for post-transplant bone mineral disease and fractures, particularly those with prolonged exposure to steroids. Lastly, post-transplant anemia may be more severe in menstruating women.^41,43^

### Role of Psychosocial Factors in Sex and Gender Disparities in Kidney Transplantation

Women probably have less knowledge and awareness of transplantation being an effective treatment for kidney failure, potentially due to a lack of discussion with their health care providers. Women may also perceive greater risks associated with kidney transplant surgery and the use of immunosuppressive medications. Additionally, the social support received from family and friends may be inadequate. However, one important positive aspect is better compliance among women, which may lead to improved kidney allograft outcomes.^44^

The role of males as primary breadwinners may restrict their interest in kidney donation due to the potential loss of productive income and valuable time. Conversely, females, who often assume caregiver roles, are more likely to donate organs. Additionally, females are inclined to exhibit greater empathy and stronger altruistic values, such as volunteering and helping others.^41,45^

Disparities in educational and socioeconomic status among women in disadvantaged communities can significantly impact their access to health care, CKD management, kidney transplantation, and allograft outcomes. Transportation issues, financial constraints, and limited resources can hinder timely referral for transplantation, completion of the evaluation process, and successful kidney allograft outcomes. Also, the perception of health care providers may add to the disparity. A survey of over 500 dialysis staff revealed a general lack of awareness regarding gender disparities in access to kidney transplantation and a lack of understanding of the contributing factors. Among those who acknowledged the existence of gender disparities, the identified contributing factors included family obligations, financial difficulties, lack of social support, fear of procedures, and concerns about physical body image.^46^

### Role of Cultural Factors in Sex and Gender Disparities in Kidney Transplantation

Health care providers may, either consciously or unconsciously, perceive female patients as frailer and at a higher risk for complications from kidney transplant surgery and immunosuppression. Use of objective assessment of frailty such as Fried assessment index, rather than subjective assessment by health care workers, will improve access to kidney transplants for females and elderly. Female patients are also more likely to report both minor and major health issues, leading to lower self-reported health status and influencing providers’ perceptions of their overall health. This can affect the perceived benefits of transplantation for female patients. Generally, females tend to exhibit greater risk aversion and may be less proactive in advocating for themselves when seeking a kidney transplant. Additionally, they may be more inclined to donate their kidneys to avoid disrupting the economic stability of families where men are the sole breadwinners. Women may recognize kidney donation as a nurturing role toward their husbands or children.^47,48^

## TRANSGENDER POPULATION

Transgender population may undergo gender-affirming surgeries to reduce gender dysphoria, such as mastectomy, breast augmentation surgery, facial surgeries, and urogenital surgeries (phalloplasty and vaginoplasty). Urogenital surgeries that involve manipulation of the urethra may increase the risk of post-transplant urethral strictures, recurrent urinary tract infections, and fistulas.^49^ As this is a new, evolving field, it is important to discuss gender-affirmative surgeries, both pretransplant and postkidney transplant, in detail.

Hormonal therapies can influence post-transplant immunosuppressive medications and transplantation surgery. Ethinyl estradiol increases the risk of venous thromboembolism and should be withheld 4 weeks prior to and after kidney transplant surgery. Estrogen may increase tacrolimus levels, and antiandrogens like spironolactone may cause hyperkalemia in the setting of kidney dysfunction. Testosterone can influence in multiple ways; it can increase alopecia along with tacrolimus, cause acne that can be exacerbated by steroids, and increase erythropoiesis, leading to post-transplant erythrocytosis.^48^

Transgender populations are at an increased risk for psychiatric illness such as anxiety, substance abuse, and depression. This can lead to medication nonadherence and increased allograft rejection rates. Hence, it is essential to have a psychiatrist in the transplantation team for detailed mental evaluation prior to and after a kidney transplant.^50^

### Future Perspectives

**Figure 1.**
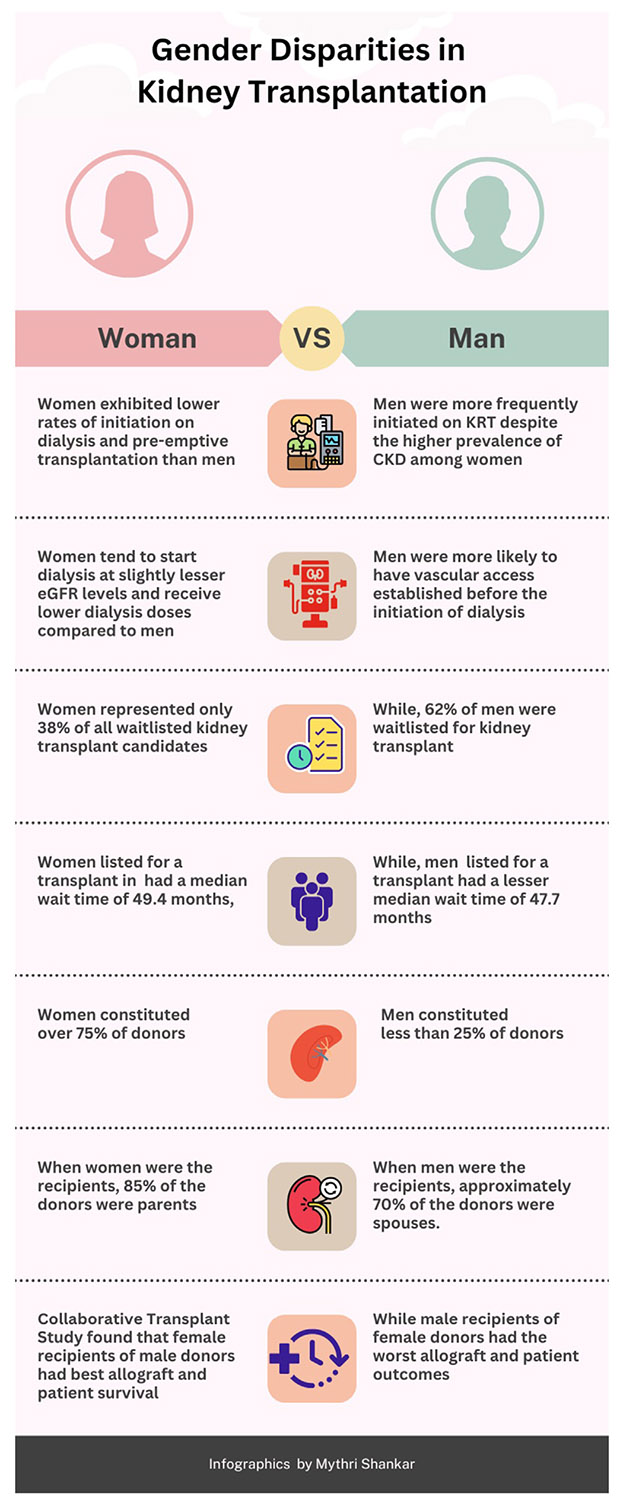
Sex and gender disparities in kidney transplantation.^7,29,36,48^ Abbreviations: eGFR, estimated glomerular filtration rate; KRT, kidney replacement therapy.

Figure 1 summarizes the sex and gender disparities in kidney transplantation. One of the top priorities should be development of an equitable deceased donor allocation system that prioritizes highly sensitized patients. Paired kidney exchange program should be strengthened locally and regionally. The process should be smooth, adaptable, and transparent.

This requires a systemic overhaul, including the creation of an independent multidisciplinary program focused on promoting, advocating, and enhancing the concept of gender equality in organ donation.

Regional and global educational workshops are imperative to raise awareness of gender-specific issues within families and households. Furthermore, the implementation of financial neutrality, modeled after the National Living Donor Assistance program in the United States, by governments to eliminate economic and financial disincentives that serve as barriers to living donation by men.

Involvement of key stakeholders and policymakers in the transplantation and donation sector is essential for establishing realistic short- and long-term objectives to bridge the gender gap. It is important to develop a unified vision and clear consensus on gender equality goals for access to both living and deceased donor transplantation across countries within the region. Moreover, implementing a culturally sensitive living donor champion program that actively involves patient parties will help align these goals.

## CONCLUSION

A regional database capturing elements of gender bias through the journey of the patients would help mitigate gender disparity. Future research should thoroughly evaluate and investigate the underlying causes of observed gender inequalities, facilitating the development and implementation of targeted interventions to address and mitigate these disparities.^5^

## Figures and Tables

**Table 1.** Sex and Gender Disparities in Kidney Allograft Outcomes and Patient Survival

Factor	Description	Influence on Graft Survival	Study Findings
Immune response	Females tend to have a more robust humoral and cellular immune response, which may increase rejection rates.	Lower short-term graft survival in females.	Varied across studies; generally shows higher rejection rates in females.^21-23^
Hormonal effects	Estrogen may protect against ischemia-reperfusion injury, potentially improving outcomes for female recipients.	Better long-term survival in females.	Supported by experimental models and clinical study.^24,25^
Nephron mass disparity	Female donors typically have less nephron mass compared to male recipients, potentially leading to reduced kidney function post-transplant.	Higher risk of graft failure in male recipients of female kidneys.	Findings from the Collaborative Transplant Study and other cohorts.^26,27^
Biological age effects	The effect of donor and recipient age on graft outcomes, with hormonal influence playing a significant role in varying immune responses.	Age-specific variations: younger females have a higher risk of graft failure; older females tend to have better outcomes than their male counterparts.	Shown in data analyses from multiple large registries.^28,29^
Socioeconomic factors	Socioeconomic status impacts access to transplant and post-transplant outcomes, with females from lower socioeconomic backgrounds experiencing worse outcomes.	Women from low-income areas have higher graft loss rates.	Studies show significant socioeconomic impact on graft survival.^30^

## CLINICAL SUMMARY

- Despite a higher prevalence of kidney disease among females, males are more likely to receive dialysis and deceased donor kidney transplants, while fewer females are refered for transplantation and places on kidney transplant waitlist
- Females are more likely to be living kidney donors, especially for spousal transplants, highlighting their caregiving roles.
- Graft survival outcomes vary between sexes due to biological differences, including immune reactions and hormonal effects, leading to conflicting findings across studies.
- Psychosocial and socioeconomic factors, such as limited knowledge, social support, and cultural factors, further exacerbate disparities in access to kidney transplantation for females.
